# Breakfast Macronutrient Composition Influences Thermic Effect of Feeding and Fat Oxidation in Young Women Who Habitually Skip Breakfast

**DOI:** 10.3390/nu8080490

**Published:** 2016-08-10

**Authors:** Brianna L. Neumann, Amy Dunn, Dallas Johnson, J. D. Adams, Jamie I. Baum

**Affiliations:** 1 Department of Food Science, University of Arkansas, 2650 North Young Avenue, Fayetteville, AR 72704, USA; blneuman@email.uark.edu (B.L.N.); amychristinedunn@gmail.com (A.D.); DJOHNSON4@uams.edu (D.J.); 2 Department of Health, Human Performance and Recreation, University of Arkansas, HPER 321, Fayetteville, AR 72701, USA; jxa014@uark.edu

**Keywords:** protein, breakfast, energy expenditure, appetite, thermic effect of feeding

## Abstract

The purpose of this study was to determine if breakfast macronutrient composition improved thermic effect of feeding (TEF) and appetite after a one-week adaptation in young women who habitually skip breakfast. A randomized, controlled study was conducted in females (24.1 ± 2 years), who skip breakfast (≥5 times/week). Participants were placed into one of three groups for eight days (*n* = 8 per group): breakfast skipping (SKP; no breakfast), carbohydrate (CHO; 351 kcal; 59 g CHO, 10 g PRO, 8 g fat) or protein (PRO; 350 kcal; 39 g CHO, 30 g PRO, 8 g fat). On days 1 (D1) and 8 (D8), TEF, substrate oxidation, appetite and blood glucose were measured. PRO had higher (*p* < 0.05) TEF compared to SKP and CHO on D1 and D8, with PRO having 29% higher TEF than CHO on D8. On D1, PRO had 30.6% higher fat oxidation than CHO and on D8, PRO had 40.6% higher fat oxidation than CHO. SKP had higher (*p* < 0.05) fat oxidation on D1 and D8 compared to PRO and CHO. There was an interaction (*p* < 0.0001) of time and breakfast on appetite response. In addition, CHO had a significant increase (*p* < 0.05) in PP hunger response on D8 vs. D1. CHO and PRO had similar PP (postprandial) glucose responses on D1 and D8. Consumption of PRO breakfast for 8 days increased TEF compared to CHO and SKP, while consumption of CHO for one week increased PP hunger response.

## 1. Introduction

Obesity is a worldwide epidemic that continues to grow [1]. Added weight is a risk factor for a number of health concerns such as type 2 diabetes, hypertension, and heart disease, however the risk of developing a chronic health condition is amplified when weight gain occurs in early adulthood [2,3,4,5]. Thus, new approaches to reduce or prevent weight gain in this age group are essential for preventing the onset of obesity and chronic disease later in life.

Breakfast is often cited as the most important meal of the day for children [6,7], but this is also true for adults. Breakfast skipping is associated with an increased risk of weight gain and obesity in young adults [8,9] as well as elevated cholesterol levels, overeating, and poor blood glucose control [6,10]. Yet, nearly 40% of American adults skip breakfast on any given day [11], despite the proven health benefits associated with eating breakfast such as increased feelings of fullness, reduced post-meal cravings [12,13,14,15,16,17], improved body composition [18], and a decreased incidence of overweight and obesity [6,19]. However, due to the lack of long-term randomized controlled trials, a strong link between breakfast skipping and health risk has not been established [8,16]. 

Thermic effect of feeding (TEF) is a potential target for the treatment of obesity since it contributes to postprandial energy expenditure and can be influenced by the macronutrient composition of the diet [20,21,22,23,24]. Meals higher in protein have a greater impact on TEF than carbohydrates [21,23,24], by increasing TEF by up to 20% [25]. Recent research has also found that increasing protein consumption (21–30 g protein) at breakfast compared to a standard cereal-based breakfast (containing 11–15 g protein) may increase subjective feelings of fullness and satiety throughout the day [26,27] and decrease caloric intake at lunch [27]. In addition, overweight women consuming sources of protein for breakfast five times a week for eight weeks lost 65% more weight and reduced their waist circumference by 83% more than those participants eating a carbohydrate-based breakfast [26]. Protein-based breakfasts positively affect postprandial blood glucose homeostasis, which is strongly associated with a lower risk of type 2 diabetes, hypertension, and cardiovascular disease. Healthy participants as well as individuals with type 2 diabetes both respond positively to high protein breakfasts, resulting in favorably altered biomarkers including reduced HbA1C%, postprandial glucose, postprandial insulin and lower systolic blood pressure [28,29,30,31]. However, most studies examining the effect of breakfast macronutrient composition are acute interventions, examining the effect of protein on TEF, appetite and glycemic response after one test meal [17,18,31]. 

To our knowledge, the adaptive response of short-term, daily breakfast consumption and composition has not been explored. Therefore, the purpose of this study was to determine if breakfast macronutrient composition improved TEF and appetite after a one-week adaptation in young women who habitually skip breakfast. 

## 2. Methods

### 2.1. Participants

Women, ages 11–36, were recruited to participate in this study. Participants were recruited through the university daily newsletter, social media, and flyers. All potential participants underwent an initial phone screening to determine if they met study qualifications. Participants enrolled in the study were self-reported habitual breakfast skippers (skipping breakfast defined as not eating before 10 a.m. [7] ≥5 days/week). Women who smoked, had dietary restrictions, were actively trying to lose weight/restricting eating, had lost weight in the last 6 months, were taking medication (excluding hormonal birth control), were athletes or participated in regular physical activity > 3 times per week, had any pre-existing metabolic conditions (e.g., type 1 or 2 diabetes) that prevented them from consuming the test breakfasts were excluded from the study. Forty women were selected to participate in the study after and signed consent forms. However, only twenty-four women completed the study: sixteen participants dropped out of the study before the first intervention day due to either scheduling conflicts (*n* = 5) or failure to appear for the first study day (*n* = 11). There was no significant difference in BMI, ethnicity, or age or those who dropped out of the study vs. those who completed the study. Participants were not informed about the group they were assigned to before the first study day. In addition, since only one participant reported to the metabolic lab per day, they were not aware of the other intervention groups. Ethical approval for the study was obtained from the Institutional Review Board at the University of Arkansas (Fayetteville, AR, USA). Written consent was obtained from all participants prior to starting the study. The ethical approval number for this study is #14-02-486. 

### 2.2. Study Design 

Participants (*n* = 24) were assigned to one of three dietary interventions using a controlled, randomized design: protein-based breakfast (PRO; *n* = 8), carbohydrate-based breakfast, (CHO; *n* = 8) or breakfast skipping (SKP; *n* = 8). To determine sample size, a power analysis was conducted using a two-tail test and alpha error of 5% to give a statistical power of 80.7%. Participants were not stratified prior to randomization. All participants completed two visits to the laboratory with seven days between visits. Participants were instructed to fast overnight and refrain from strenuous physical activity the day before testing. On study day 1 (D1), participants arrived at the Food Science Department at the University of Arkansas at 7:30. Upon arrival, body weight and height were measured. Fasting blood glucose levels, resting energy expenditure (REE), and baseline appetite assessments were also measured. Participants then continued to skip breakfast or were provided with either a protein- or carbohydrate-based breakfast. Following the REE measurement, participants eating breakfast were given 15 min to consume the entire test breakfast. Glucose and appetite assessments were collected at 15, 30, 60, 90, and 120 min postprandial (PP). Thermic effect of feeding (TEF) was calculated at 30, 60, 90, and 120 min following breakfast. At the end of D1, participants were provided with six breakfast meals corresponding to the breakfast group to which they were assigned. Since breakfast has been defined as the first meal of the day; eaten before or at the start of daily activities (e.g., errands, travel, work, etc.) and within two hours of waking; typically no later than 10:00 a.m. [7], participants were instructed to consume each breakfast according to these guidelines for the following six days. Participants were also required to complete three, 24-h food intake records (two weekdays and one weekend; including all meals, snacks and beverages). Participants were given a tutorial on how to complete the food intake record as well as an example to take home with them. The days for completing the food intake records were preassigned before the participants left the research facility. Participants were also required to maintain their typical physical activity level throughout the intervention period and record all physical activity in a designated section of the 24-h food intake record. On day 8 (D8), participants returned to the Food Science Department at the University of Arkansas in a fasted state to repeat the same study protocol as D1. 

### 2.3. Test Breakfasts 

Participants were assigned to one of three test breakfasts, which they consumed each day of the intervention period: a carbohydrate-based (CHO) breakfast, a protein-based (PRO) breakfast, or they continued to skip (SKP) breakfast. The CHO breakfast consisted of 1 English muffin (57 g), yogurt (170 g), cream cheese (17 g), and water (227 mL). The PRO breakfast consisted of a proprietary breakfast sandwich (145 g), Greek yogurt (150 g), and water (227 mL). Both test breakfast were similar in kilocalories and controlled for fat and fiber (Table 1). The SKP group was provided water (227 mL). All foods were purchased commercially (refer to Table S1).

### 2.4. Anthropometric Measurements

Body height was measured to the nearest 0.01 cm using a stadiometer (Detecto, St. Louis, MO, USA) with participants barefoot, in the free-standing position. Body weight was measured in the fasting state with participants barefoot to the nearest 0.01 kg using calibrated balance scales (Detecto). BMI was calculated as weight (kg) divided by height (m) squared. Body composition, including fat-free mass (FFM), was assessed by dual energy X-ray absorptiometry (DEXA; Lunar Prodigy, GE Healthcare, Madison, WI, USA) in the Human Performance Laboratory at the University of Arkansas. 

### 2.5. Energy Expenditure and Substrate Oxidation

Resting energy expenditure (REE; kcal/min) was measured using indirect calorimetry with a TrueMax 2400 metabolic cart and ventilation hood (Parvomedics, Sandy, UT, USA) at 0, 30, 60, 90, and 120 min. REE measured by indirect calorimetry under standard conditions provides information at rest in the form of oxygen consumption (VO_2_), carbon dioxide production (VCO_2_), and respiratory exchange ratio (RER), see Equation (1) [32]. 


RER = VCO_2_/VO_2_ (1)

This method has been validated by Cooper et al. [32]. The metabolic cart was calibrated to account for change in environmental conditions at the beginning of each study day. REE was measured in 30 s increments during a 20 min rest period while participants were in the supine, reclined position. Only the last 15 min were used for analysis from each time point and the first five minutes were discarded [20]. TEF (kcal/min) for each time point was determined by assessing the difference between REE at time 0 and times 30, 60, 90, and 120 min (e.g., TEF = REE_120_ − REE_0_). Both REE and TEF were controlled for FFM. To control for FFM, REE and TEF were divided by total FFM (FFM in kg as determined by DEXA). Respiratory quotient (RQ), VO_2_ (mL/min), VCO_2_ (mL/min) were calculated by the TrueMax 2400 metabolic cart software using the rate of oxygen inhalation compared with carbon dioxide exhalation. Substrate oxidation rates were also calculated by the TrueMax 2400 metabolic cart software (version 4.3.4, Sandy, Utah, USA) using the RQ values calculated by Equation (2).


(RQ = CO_2elminated_ − O_2consumed_). (2)

### 2.6. Appetite and Palatability Ratings

Appetite and palatability were assessed using a traditional 100-mm visual analog scale (VAS) [33] with opposing anchors (e.g., “extremely hungry” or “not hungry at all”) at time points 0, 15, 30, 60, 90, and 120 min. Questions consisted of: “how hungry do you feel at this moment”, “how full do you feel at this moment”, “how strong is your desire to eat this moment” and “how much food do you think you can eat at this moment”. Appearance (“how much do you like or dislike appearance of the breakfast foods”) and palatability (“how much do you like or dislike the smell and taste of the breakfast foods”) of test breakfasts were assessed during breakfast consumption on D1 and D8 using a traditional 100-mm VAS with opposing anchors “dislike extremely” or “like extremely”. Participants were asked to place an “X” on the 100-mm VAS in the place that pertained to their perceived appetite feelings at each time point.

### 2.7. Blood Glucose Measurements

One 0.1 mL blood sample was collected in a capillary tube (Health Management Systems, Corp., Plano, TX, USA) via finger stick at 0, 15, 30, 60, 90, and 120 min PP. A different finger was used for each finger stick, so that the same finger was not used more than once during each study day. Blood glucose levels were determined using a Lifescan One Touch UltraSmart System (New Brunswick, NJ, USA). Each sample was measured in duplicate from the same capillary tube and the average was used in analysis [20]. 

### 2.8. Dietary Assessment

The energy and macronutrient composition of test breakfast meals and the three, 24-h food intake records were analyzed using Genesis R & D nutrient analysis software (version 9.10.2, ESHA Research, Salem, OR, USA). 

### 2.9. Statistical Analysis 

Summary statistics were calculated for all data (sample means and sample standard error of mean). Two-sample independent t-test were used to analyze breakfast palatability and appearance. Two-factor analysis of variance (ANOVA) was used to determine diet × time interaction for appetite ratings, glucose levels, TEF, RQ, VO_2_, VCO_2_, and substrate oxidation. If differences were found, two-factor, repeated measure ANOVA was used to determine differences. Where significance was found, the Bonferroni correction was applied and two-sample independent *t*-test was used to determine the degree of significance. Net incremental area under the curve (niAUC) was calculated for appetite ratings, TEF, RQ, substrate oxidation, and glucose levels. One-factor analysis of variance (ANOVA) was used to compare demographics, dietary intake, REE, and niAUC of diet groups for appetite ratings, glucose levels, TEF, and substrate oxidation. When significance or a trend was found, a two-sample independent *t*-test was used to determine the degree of significance or trend. Paired *t*-test was used to determine within diet differences for appetite ratings, glucose levels, REE, TEF, and substrate oxidation. We controlled for age throughout the analyses, since there was a significant difference in age between SKP and PRO and CHO. All results reported as means ± SEM. All data was analyzed using GraphPad Prism Software version 6.0 (GraphPad Software, La Jolla, CA, USA). *p* < 0.05 was considered statistically significant. 

## 3. Results

### 3.1. Participant Characteristics

Participant demographics are presented in Table 2. There was no significant difference in height, weight, BMI, body fat percentage, or FFM between diet groups. Age was significantly higher in SKP (*p* < 0.05) compared to PRO and CHO. There was no significant different in height, weight, or BMI between D1 and D8 (refer to Table S2). 

### 3.2. Energy Expenditure and Substrate Oxidation

**Figure 1:**
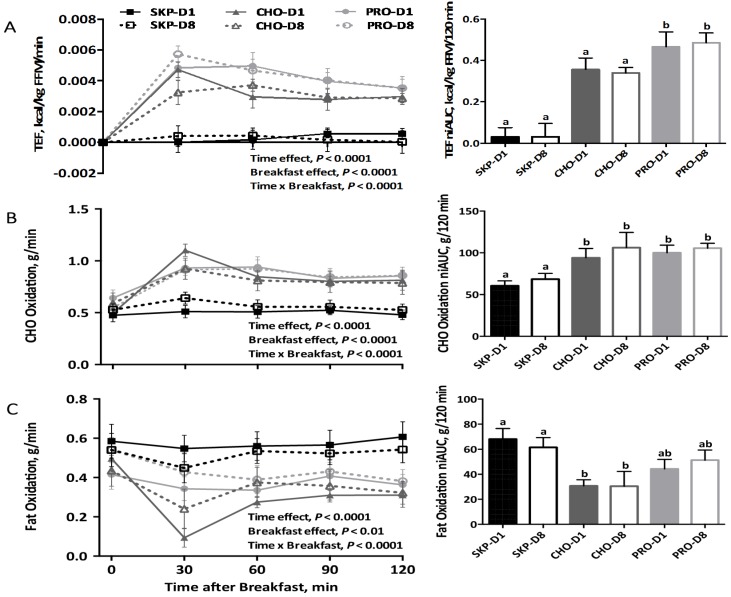
Energy expenditure and substrate oxidation following a PRO- or CHO-breakfast or continued breakfast skipping. Data are expressed as means ± SEMs; SKP *n* = 8, PRO *n* = 8, CHO *n* = 8. (**A**) Postprandial energy expenditure (TEF) controlled for fat-free mass over time per breakfast group and niAUC for TEF for each breakfast group; (**B**) Carbohydrate oxidation over time per breakfast group and niAUC for carbohydrate oxidation for each breakfast group; (**C**) Fat oxidation over time per breakfast group and niAUC for fat oxidation for each breakfast group. Labeled bars without a common letter differ, *p* ≤ 0.05. SKP, breakfast skipping; CHO, carbohydrate-based breakfast; PRO, protein-based breakfast; FFM, fat-free mass.

There was no difference in REE between participants (Figure S1). EE and substrate oxidation are presented in the line graphs (individual time points) and bar graphs (niAUC) in Figure 1. Overall, there was a significant (*p* < 0.0001) effect of time, breakfast, and breakfast over time on TEF, carbohydrate oxidation, and fat oxidation. There was no difference between D1 or D8 for TEF, carbohydrate oxidation, and fat oxidation. However, participants consuming PRO had significantly higher (*p* < 0.05; ~21 kcal/120 min) niAUC for TEF compared to CHO (~15 kcal/120 min) and SKP (~1.5 kcal/120 min. There was a significant effect (*p* < 0.05) of consuming breakfast on fat and carbohydrate oxidation, with no effect of breakfast type. In addition, PRO had 30.6% higher fat oxidation (44.3 g/120 min) than CHO (30.7 g/120 min) on D1 and PRO had 40.6% higher fat oxidation (51.3 g/120 min) than CHO (30.5 g/120 min) on D8. The results for RQ, VO_2_, and VCO_2_ are presented in Table S3.

### 3.3. Appetite and Palatability Ratings

**Figure 2:**
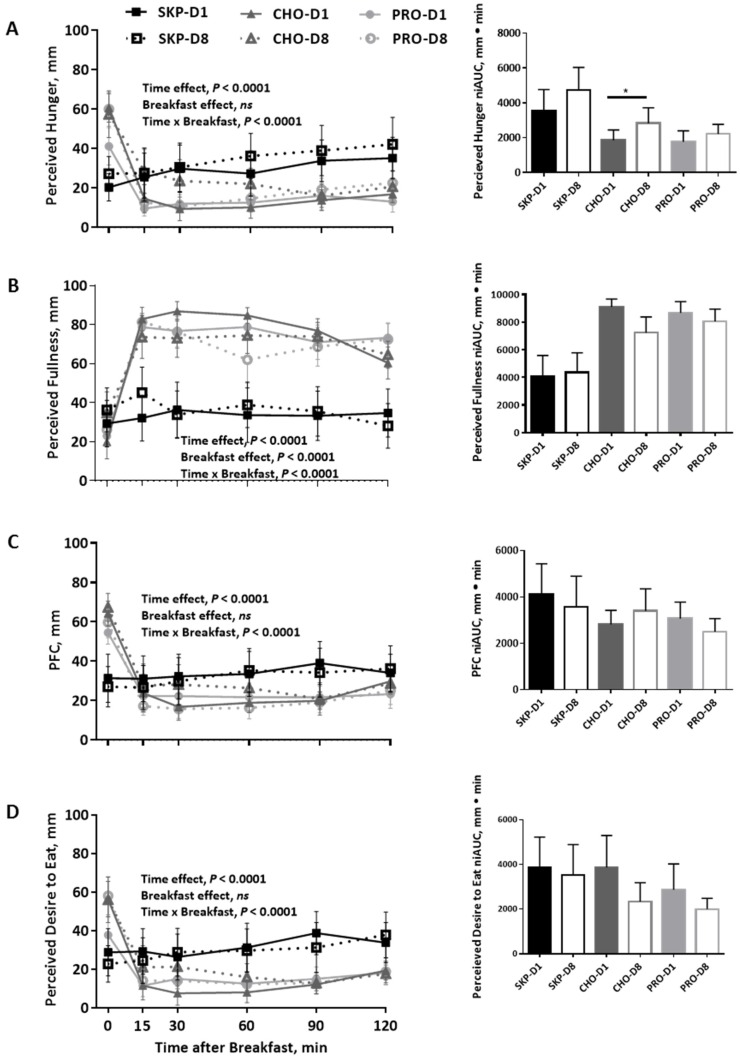
Ratings of appetite two hours postprandial (PP) following a PRO- or CHO-breakfast or continued breakfast skipping using visual analog scales. Data are expressed as means ± SEMs; SKP *n* = 8, PRO *n* = 8, CHO *n* = 8. (**A**) Perceived hunger over time and net incremental area under the curve (niAUC) for perceived hunger for each breakfast group; (**B**) Perceived fullness over time and niAUC for perceived fullness for each breakfast group; (**C**) Prospective food consumption (PFC) over time and niAUC for PFC for each breakfast group; (**D**) Perceived desire to eat over time and niAUC for perceived desire to eat for each breakfast group. * *p* ≤ 0.05. SKP, breakfast skipping; CHO, carbohydrate-based breakfast; PRO, protein-based breakfast.

Results for perceived hunger, perceived fullness, prospective food consumption (PFC), and perceived desire to eat are presented in the line graphs (individual time points) and bar graphs (niAUC) in Figure 2. For each appetite response, there was an effect of time and breakfast over time (*p* < 0.0001 for each). There was a significant effect of breakfast consumption, not breakfast type, on perceived fullness (*p* < 0.0001). There was no difference in appetite response between D1 and D8 within diets. However, participants following the CHO breakfast reported increased hunger following consumption of the CHO breakfast on D8 vs. D1 (*p* < 0.01). There was no difference in appearance or palatability between the CHO and PRO breakfast (Table 1). 

### 3.4. Blood Glucose

**Figure 3:**
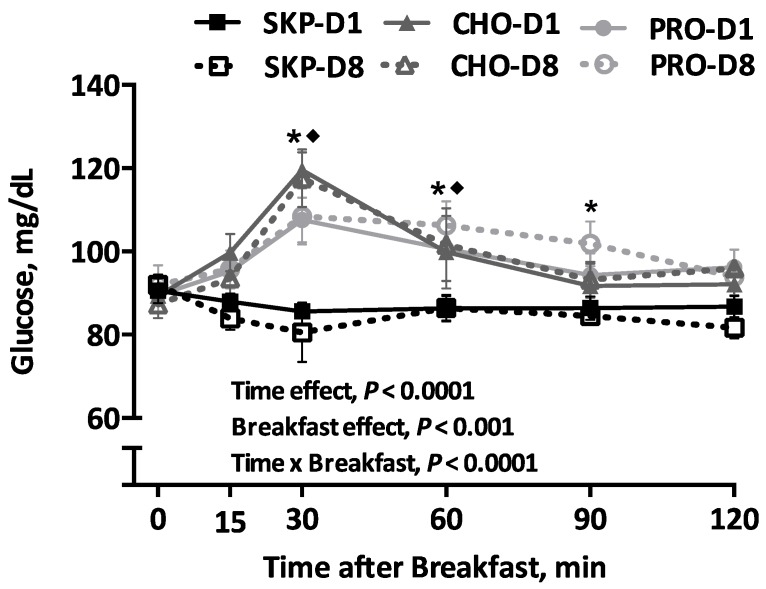
Changes in glucose response over time following a PRO- or CHO-breakfast or continued breakfast skipping. Data are expressed as means ± SEMs; SKP *n* = 8, PRO *n* = 8, CHO *n* = 8. Glucose response to the test breakfasts over time. * Difference between pooled (D1 + D8) SKP and pooled PRO, *p* ≤ 0.05; ◆ difference between pooled SKP and pooled CHO, *p* ≤ 0.05. SKP, breakfast skipping; CHO, carbohydrate-based breakfast; PRO, protein-based breakfast.

The results for blood glucose are presented in Figure 3. There was no difference in fasting blood glucose between groups and study days. There was a main effect of time, breakfast and breakfast over time (*p* < 0.0001) on blood glucose levels. CHO and PRO lead to greater increase in glucose values compared to SKP at 30 and 60 min PP (*p* < 0.01). However, PRO had a 10% lower glucose levels compared with CHO at 30 min PP. There was no difference in niAUC values between PRO or CHO breakfasts or between D1 and D8 within diets. 

### 3.5. Ad Libitum Dietary Assessment

Average daily energy intake is provided in Table 3. There was no significant effect of breakfast consumption or breakfast skipping on total energy (kcal) intake. However, participants consuming CHO had 25% lower energy intake compared to SKP and 33% lower energy intake compared to PRO.

## 4. Discussion

To our knowledge this is the first study to examine the effect of breakfast macronutrient composition over an eight-day adaptation period on PP energy metabolism, appetite response, glucose response, and 24-h food intake in breakfast skipping females. Breakfast consumption increased TEF compared to SKP and consumption of PRO increased TEF compared to consumption of CHO. Breakfast consumption also increased PP substrate oxidation, with a trend for PRO breakfast to increase fat oxidation compared to CHO. The macronutrient content of the breakfasts did not impact overall glucose response, however PRO had a lower glucose peak at 30 min PP and a slower return to baseline values compared to CHO. There was no effect of the eight-day adaptation period on energy metabolism, substrate oxidation, glucose or appetite response, with the exception of hunger. CHO intake over the eight-day adaptation period significantly increased PP hunger. However, despite this increase in PP hunger, CHO consumed ~400 less calories compared to SKP and ~500 less calories compared to PRO (although not a significant difference). Collectively, this study demonstrates that daily consumption of a breakfast higher in protein for one week increases TEF and fat oxidation compared to a carbohydrate-based breakfast, and that breakfast consumption, in general, has more benefits related to energy expenditure than breakfast skipping in the short-term. 

Breakfast is often recognized as the most important meal of the day [9,20,34]. However there is debate as to what defines the ideal breakfast meal [34], in addition to a lack of strong evidence to define which nutrients should be represented at breakfast [34]. A recent commentary published by the American Academy of Nutrition and Dietetics suggests that protein-containing foods (e.g., eggs, lean meat and low-fat dairy products) should be included in breakfast meals [34]. Literature supports diets higher in protein aid in the treatment of chronic, metabolic diseases such as obesity, type 2 diabetes and heart disease and have been shown to increase EE, improve satiety, regulate glycemic control and improve body composition (reviewed in [35,36,37,38]). However, the role of breakfasts higher in protein on metabolic health still needs to be defined. 

The relationship between protein intake and increased TEF is well-established [20,23,31,39,40]. However, very few studies have examined the impact of habitual breakfast consumption on TEF. Furthermore, most protein intake studies conducted have use isolated protein sources, often consumed in liquid form, as the intervention rather than protein as part of a complete meal [31,40,41,42]. For example, Acheson et al. [31] administered whey-, casein-, soy-protein, and carbohydrate-based beverages for a breakfast meal, and demonstrated that the protein beverages, independent of protein source, increased TEF to a greater extent than the carbohydrate beverage in young men over a five-hour period [31]. In another study, both male and female young adults consumed either a high protein, low carbohydrate shake (30% energy from protein) or a low protein, high carbohydrate shake (5% energy from protein) over the course of 12 weeks [42]. At the end of the intervention period, the participants consuming the high carbohydrate shake had a significant reduction in TEF compared to those consuming the high protein shake and compared to baseline values, which is in agreement with the findings from this study. Although we observed an increase in TEF following the breakfast meals on both D1 and D8, we do not know if this effect would last throughout the day. Recent studies suggest that even though breakfast consumption may increase TEF and physical activity in the morning, at the end of the day, there was no difference observed in energy expenditure when compared to those who did not eat breakfast [43,44]. Another study by Arciero et al. [24], found that after fifty-six days of diet adaptation, subjects receiving a high protein breakfast had higher TEF compared to baseline control values. However, this measurement was taken after adaptation to either three or six high-protein meals per day, therefore the results are not reflective of breakfast, alone. In contrast, in the current study, there were no differences in D1 and D8 in TEF and substrate oxidation within groups. This could be due to the short adaptation period and if the intervention had been longer, we may have seen an effect of adaptation.

A majority of the breakfast literature is composed of acute meal studies, which make it difficult to make conclusions about the longer-term effects of breakfast interventions [11,16,17,18,20]. Interestingly, just consuming breakfast in the morning has been shown to only transiently suppress appetite (i.e., 1–5 h) compared to skipping breakfast, without any difference over the remaining-hour period [9]. This further supports the importance of protein consumption within the breakfast meal. Several acute studies have examined the effect of breakfast macronutrient composition on appetite regulation and energy intake. Leidy and Racki [17] demonstrated consuming breakfast increases feelings of fullness in breakfast skipping adolescents and breakfasts higher in protein decreases appetite to a greater extent than normal protein breakfasts. In another longer-term study (12-weeks), examining the impact of a high-protein breakfast vs. a high-carbohydrate breakfast on appetite response, found an increase in 24-h PP fullness and satiety following consumption of the high-protein breakfast for one-week compared to the high-carbohydrate breakfast, however this difference was not detected at the end of the 12-week intervention [42]. Although 24-h appetite measurements were not taken in the current study, there was a suppression of appetite for two hours following breakfast consumption on both D1 and D8 of the intervention, with no impact of breakfast macronutrient composition. These results are further supported by Leidy et al. [45], who found a significant effect of breakfast consumption on appetite suppression in breakfast-skipping, late-adolescent females, but no effect of breakfast macronutrient composition after seven days of breakfast consumption. However, it should be noted, although not significant, that in this study average daily calorie intake decreased by ~400 calories in CHO compared to SKP and ~500 calories compared to PRO. This could be due to the lower BMI and body fat percentage of the CHO and may have reached significance if the study had higher power. Another possible explanation could be variations in physical activity throughout the week, however we did not collect this information.

There is an association between habitual breakfast skipping, higher BMI, and an increased risk of chronic disease [9]. Therefore, it is often argued that breakfast consumption could be an effective weight loss strategy since eating breakfast is often associated with reduced caloric intake and increased nutrient intake throughout the day when compared to habitual breakfast skippers [4,8]. In this study, although breakfast consumption increased feelings of fullness and decreased feelings of hunger, there was no effect of breakfast consumption or breakfast composition on 24-h energy intake. This is supported by Leidy and Racki [17] who found that breakfast consumption and breakfast composition influenced energy intake at lunch, however total 24-h energy intake was not different between groups. 

Consumption of a high-protein diet has been linked to improved glycemic response, in both the short- [12,20,31] and long-term [30,37,46]. In this study, there was no effect of breakfast composition or breakfast adaptation on PP glycemic response. However, these results are consistent with findings from Alwattar et al. [15], who found no difference in PP glycemic response between a high protein and high carbohydrate breakfast over time. 

### Strengths and Limitations

One of the strengths of this manuscript is the adaptation period of breakfast consumption in breakfast skipping young women. Although short, at only one week, most breakfast interventions only examine the acute, postprandial response. Although Martens et al. [42] found an effect of increased dietary protein intake on energy metabolism over a 12-week study, their subjects were not habitual breakfast skippers and consumed a higher protein vs. higher carbohydrate diet. There are several limitations to this study. This study was conducted as a parallel design and not a randomized, crossover design, which would have strengthened our findings. We relied on self-reports of habitual breakfast skipping and did not further validate this by asking if they ate breakfast foods after 10:00 a.m., our cut off time for breakfast consumption. We did not collect plasma or urine samples, which limited the analysis we could perform (e.g., hormones associated with appetite, protein oxidation, glucose tolerance, insulin sensitivity). We used finger sticks for blood glucose analysis, which has limited accuracy, and may be why no difference in postprandial blood glucose response was observed between PRO and CHO. However, we have previously published this method [20] for assessment of blood glucose in children. We also did not control for menstrual cycle. In addition, we used self-reported food intake, which is not always an accurate measure of food intake [47]. We did not have participants record food intake on the same days of the intervention period, instead we allowed participants to complete food intake records for the two week days and one weekend day of their choice. Finally, it would have been useful to have a habitual breakfast eater group, to determine the magnitude of postprandial changes in breakfast skippers vs. eaters. 

## 5. Conclusions

In conclusion, breakfast consumption (PRO and CHO) decreased PP hunger and increased fullness compared to SKP, with no effect of breakfast composition. However, there was no effect of breakfast consumption on prospective food consumption and perceived desire to eat. There was an increase in TEF with PRO compared to CHO. In addition, consumption of CHO for eight days resulted in an increased hunger response, however this did not impact calorie intake. There was no impact of the eight-day adaptation period on any other outcomes. Taken together, these data suggest that increasing protein at breakfast has beneficial effects on TEF in habitual breakfast skipping women in the short-term, but a longer adaptation period may be needed.

## Figures and Tables

**Table 1:** Dietary characteristics of test breakfasts ^1^.

	CHO	PRO
Energy content, kJ (kcal)	1469 (351)	1464 (350)
Total protein, g	10	30
Total carbohydrate, g	59	39
Total sugars, g	27	9
Fiber, g	1	2
Total fat, g	8	8
Breakfast Appearance, mm ^2^	69 ± 4	64 ± 5
Breakfast Palatability, mm ^2^	75 ± 3	68 ± 5

^1^ Values are means ± SEM, *n* = 8 per diet. CHO, carbohydrate-based breakfast; PRO, protein-based breakfast; ^2^ Units are in millimeters (mm) according to a traditional 100-mm visual analog scale. Mean values are combined days 1 and 8 data.

**Table 2:** Participant characteristics ^1^.

	SKP	CHO	PRO
Participants, *n*	8	8	8
Age, year	27.1 ± 1.8 ^a^	21.9 ± 0.9 ^b^	23.3 ± 1.3 ^a,b^
Height, cm	168.4 ± 2.1	162.1 ± 4.1	164.9 ± 2.2
Weight, kg	78.9 ± 6.3	67.0 ± 7.0	72.6 ± 6.3
BMI	27.8 ± 2.2	26.0 ± 1.9	26.6 ± 2.1
Fat Mass, %	45.3 ± 1.6	37.4 ± 3.1	40.5 ± 3.4
Fat Free Mass, kg	45.8 ± 3.4	43.6 ± 2.3	44.5 ± 1.5
Ethnicity			
Caucasian	5	3	6
Hispanic	1	1	1
Black	1	1	1
Asian		2	
Indian	1	1	

^1^ Values are means ± SEM; represent measurements taken on D1. Labeled means in a row without a common letter differ, *p* < 0.05. SKP, breakfast skipping; CHO, carbohydrate-based breakfast; PRO, protein-based breakfast.

**Table 3:** Average daily energy and macronutrient intake during adaptation period ^1^.

	SKP	CHO	PRO
Energy Intake			
Total, kJ	8406 ± 808	6707 ± 531	8941 ± 1460
Total, kcal	2009 ± 193	1603 ± 127	2137± 349
Protein, g	88 ± 13 ^a,b^	57 ± 6 ^b^	99 ± 8 ^a^
Carbohydrate, g	234 ± 22	231 ± 19	246 ± 40
Fat, g	75 ± 9	54 ± 5	81 ± 17
Macronutrient Intake ^2^			
Protein, g	18	14	19
Carbohydrate, g	47	57	46
Fat, g	35	29	35

^1^ Values are means ± SEM. Data obtained from 3-day food records. Labeled means in a row without a common letter differ, *p* < 0.05. SKP, breakfast skipping; CHO, carbohydrate-based breakfast; PRO, protein-based breakfast; ^2^ Data expressed as percent energy of energy intake.

## Supplementary Materials

The following are available online at http://www.mdpi.com/2072-6643/8/8/490/s1, Figure S1: Resting energy expenditure (REE) at baseline. There was no significant difference in REE between dietary treatments or day 1 and 8 of the intervention period, Table S1: Brands of foods used in the dietary intervention, Table S2: BMI, Height and Weight of Participants on D1 and D8, Table S3: Postprandial metabolic variables following consumption of either CHO- or PRO- based test breakfast.
