# Supplementary material for: Breakfast Macronutrient Composition Influences Thermic Effect of Feeding and Fat Oxidation in Young Women Who Habitually Skip Breakfast
Source: Nutrients. 2016 Aug 10;8(8):490. doi: 10.3390/nu8080490 (PMC4997403; doi:10.3390/nu8080490)
Supplement: Supplementary file 1 [file nutrients-08-00490-s001.docx]

Supplementary Materials: Breakfast Macronutrient Composition Influences Thermic Effect of Feeding and Fat Oxidation in Young Women Who Habitually Skip Breakfast

Brianna L. Neumann, Amy Dunn, Dallas Johnson, J. D. Adams and Jamie I. Baum

**Figure S1.** Resting energy expenditure (REE) at baseline. There was no significant difference in REE between dietary treatments or day 1 and 8 of the intervention period.

**Table S1.** Brands of foods used in the dietary intervention.

| **Protein-Based Breakfast** | **Carbohydrate-Based Breakfast** |
| --- | --- |
| Jimmy Dean Delights turkey sausage, egg white & cheese English muffin | Thomas’ English muffin, original |
| Yoplait Greek 100 vanilla yogurt | Yoplait original vanilla yogurt |
| Nestle drinking water | Kraft Philadelphia cream cheese |
|  | Nestle drinking water |

**Table S2.** BMI, Height and Weight of Participants on D1 and D8.

|  | **Test Day 1** | | | **Test Day 2** | | |
| --- | --- | --- | --- | --- | --- | --- |
| **SKP** | **BMI** | **Height (cm)** | **Weight (kg)** | **BMI** | **Height (cm)** | **Weight (kg)** |
| 1 | 25.2 | 170.18 | 73.03 | 25.2 | 170.18 | 73.03 |
| 2 | 40.2 | 170.18 | 116.57 | 40.3 | 170.18 | 116.80 |
| 3 | 33 | 162.56 | 87.09 | 23.8 | 173.36 | 71.67 |
| 4 | 24 | 162.56 | 63.50 | 33 | 162.56 | 87.32 |
| 5 | 25.3 | 160.02 | 64.86 | 24.5 | 162.56 | 64.64 |
| 6 | 23.8 | 174.63 | 72.57 | 25.4 | 159.39 | 64.64 |
| 7 | 22.3 | 175.60 | 68.49 | 22.8 | 173.99 | 68.94 |
| 8 | 29.9 | 171.45 | 87.99 | 29.9 | 171.45 | 87.27 |
| **CHO** | **BMI** | **Height (cm)** | **Weight (kg)** | **BMI** | **Height (cm)** | **Weight (kg)** |
| 1 | 28.5 | 151 | 68.492 | 28.5 | 151.00 | 71.21 |
| 2 | 25.7 | 175.26 | 78.93 | 25.7 | 172.20 | 75.66 |
| 3 | 22.1 | 175.26 | 68.04 | 21.9 | 175.90 | 67.81 |
| 4 | 21.9 | 149.86 | 49.21 | 22 | 149.86 | 49.44 |
| 5 | 34.3 | 172.72 | 105.23 | 36.5 | 170.18 | 105.67 |
| 6 | 33.3 | 161.29 | 86.64 | 34 | 161.29 | 87.99 |
| 7 | 21.6 | 148.50 | 47.60 | 21.6 | 148.50 | 47.60 |
| 8 | 20.8 | 163.20 | 55.34 | 20.8 | 163.20 | 55.34 |
| **PRO** | **BMI** | **Height (cm)** | **Weight (kg)** | **BMI** | **Height (cm)** | **Weight (kg)** |
| 1 | 25.7 | 168.91 | 73.26 | 26 | 167.64 | 73.03 |
| 2 | 28.2 | 168 | 79.379 | 27.4 | 167.64 | 81.42 |
| 3 | 25.8 | 156.21 | 63.05 | 25.8 | 156.21 | 63.05 |
| 4 | 26.5 | 169.55 | 76.20 | 26.2 | 169.55 | 75.30 |
| 5 | 20.1 | 163.83 | 53.98 | 19.9 | 163.83 | 53.30 |
| 6 | 27.8 | 172.00 | 83.00 | 28.2 | 170.80 | 82.78 |
| 7 | 38.11 | 164.00 | 102.50 | 38.70 | 164.00 | 104.09 |
| 8 | 19.80 | 156.00 | 48.18 | 19.99 | 156.00 | 48.64 |

**Table S3.** Postprandial metabolic variables following consumption of either CHO- or PRO- based test breakfast ^1^.

| **Time Following Breakfast (min)** | **SKP** | | **CHO** | | **PRO** | | **Effect of Time** | **Effect of Breakfast Type** | **Time × Breakfast Type Interaction** |
| --- | --- | --- | --- | --- | --- | --- | --- | --- | --- |
| *n*/group | 8 | | 8 | | 7 | |  |  |  |
|  | PRE | POST | PRE | POST | PRE | POST |  |  |  |
| RQ |  |  |  |  |  |  | <0.0001 | <0.0001 | <0.0001 |
| 0 | 0.836 ± 0.019 | 0.850 ± 0.018 | 0.851 ± 0.013 A | 0.867 ± 0.023 A | 0.879 ± 0.019 A | 0.849 ± 0.018 A |  |  |  |
| 30 | 0.843 ±0.017 a | 0.876 ± 0.016 a | 0.976 ± 0.012 bB | 0.912 ± 0.017 abcB | 0.916 ± 0.015 bcB | 0.902 ± 0.019 acB |  |  |  |
| 60 | 0.843 ± 0.017 a | 0.851 ± 0.015 ab | 0.909 ± 0.015 abC | 0.901 ± 0.018 abAB | 0.916 ± 0.019 bB | 0.906 ± 0.017 abB |  |  |  |
| 90 | 0.846 ± 0.014 | 0.852 ± 0.016 | 0.902 ± 0.013 C | 0.903 ± 0.019 AB | 0.899 ± 0.017 AB | 0.894 ± 0.017 B |  |  |  |
| 120 | 0.834 ± 0.015 a | 0.847 ± 0.015 ab | 0.912 ± 0.0147 bC | 0.910 ± 0.016 bB | 0.909 ± 0.013 bAB | 0.905 ± 0.016 bB |  |  |  |
| VO_2_, mL/min ^2^ |  |  |  |  |  |  | <0.05 | <0.01 | ns |
| 0 | 4.861 ± 0.185 ab | 5.074 ± 0.144 ab | 4.685 ± 0.092 aA | 4.819 ± 0.083 abA | 5.914 ± 0.249 b | 5.610 ± 0.971 ab |  |  |  |
| 30 | 4.848 ± 0.163 | 5.128 ± 0.158 | 5.504 ± 0.113 B | 5.405 ± 0.115 AB | 5.914 ± 0.249 | 5.610 ± 0.971 |  |  |  |
| 60 | 4.887 ± 0.155 | 5.163 ± 0.123 | 5.163 ± 0.129 AB | 5.540 ± 0.091 B | 5.931 ± 0.274 | 6.033 ± 0.371 |  |  |  |
| 90 | 4.967 ± 0.182 | 5.104 ± 0.132 | 5.104 ± 0.120 AB | 5.373 ± 0.112 AB | 5.759 ± 0.179 | 5.937 ± 0.306 |  |  |  |
| 120 | 4.975 ± 0.171 | 5.081 ± 0.177 | 5.081 ± 0.110 AB | 5.358 ± 0.105 AB | 5.660 ± 0.199 | 5.802 ± 0.251 |  |  |  |
| VCO_2_, mL/min ^2^ |  |  |  |  |  |  | <0.0001 | <0.0001 | <0.0001 |
| 0 | 4.054 ± 0.147 | 4.308 ± 0.123 | 3.985 ± 0.085 A | 4.187 ± 0.157 A | 4.377 ± 0.207 A | 4.351 ± 0.303 A |  |  |  |
| 30 | 4.098 ± 0.181 a | 4.493 ± 0.153 ac | 5.377 ± 0.156 bB | 5.059 ± 0.120 bcB | 5.432 ± 0.262 bB | 5.560 ± 0.331 bB |  |  |  |
| 60 | 4.116 ± 0.151 a | 4.393 ± 0.131 a | 4.818 ± 0.141 abC | 5.005 ± 0.174 bB | 5.481 ± 0.317 bB | 5.484 ± 0.351 bBC |  |  |  |
| 90 | 4.198 ± 0.156 a | 4.351 ± 0.142 a | 4.738 ± 0.135 abC | 4.867 ± 0.181 abB | 5.188 ± 0.200 bB | 5.291 ± 0.305 bBC |  |  |  |
| 120 | 4.147 ± 0.144 a | 4.307 ± 0.171 a | 4.776 ± 0.173 abC | 4.882 ± 0.141 abB | 5.150 ± 0.213 bB | 5.265 ± 0.249 bC |  |  |  |

^1^ Values are means ± SEMs. Labeled means within a treatment column without a common upper case letter differ, *p* < 0.05. If a value within a column does not contain an upper case letter, there is no difference within the column. Labeled means within a row without a common lower case letter differ, *p* < 0.05. If a row does not contain a lower case letter, there is no difference within that row. Respiratory Quotient, RQ. ^2^ Controlled for Fat Free Mass.
